# Endoscopic full-thickness defect closure using a novel suture anchor device: a pilot survival porcine study

**DOI:** 10.1055/a-2702-4999

**Published:** 2025-10-02

**Authors:** Jiancong Feng, Yaqi Zhai, Zhenyu Liu, Enqiang Linghu

**Affiliations:** 1 Department of Gastroenterology, The First Medical Center of Chinese PLA General Hospital, Beijing, China

For gastric submucosal tumors originating from the muscularis propria, endoscopic full-thickness resection (EFTR) represents a safe and effective super minimally invasive surgery, in which secure defect closure is paramount 1 . Despite widespread adoption for closing post-EFTR transmural defects, through-the-scope clips and endoloop techniques remain inadequate for reliable full-thickness closure owing to superficial tissue grasp 2 .

To address this limitation, a novel endoscopic suture anchor device ( Fig. 1 ) that enables screwing into tissues was developed. Previously validated for mucosal defect closure after endoscopic submucosal dissection 3 4 , this device was evaluated for the first time in an in vivo porcine model for closing post-EFTR defects.

**Fig. 1:**
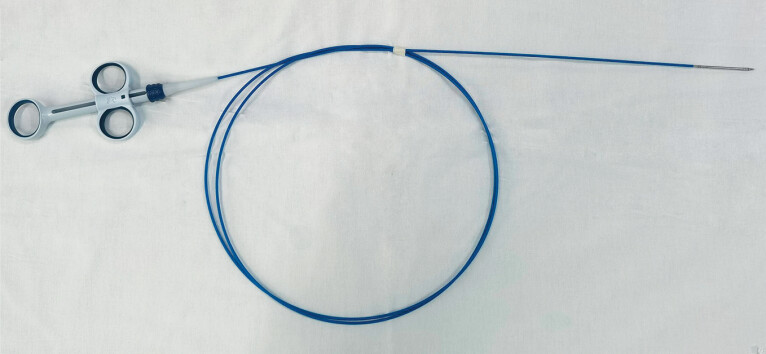
Endoscopic suture anchor device (Micro-Tech, Nanjing, China).

A 2.5 × 1.5-cm “active perforation” was created in the anterior gastric body of a porcine model to simulate a post-EFTR transmural defect. Subsequently, the suture anchor devices were employed to close the defect using the following steps ( Video 1 ).

Endoscopic suture anchor closure of gastric transmural defect in porcine model.Video 1

Six suture anchors pre-threaded with a nylon suture were deployed sequentially via the endoscopic working channel (GIF-Q260J; Olympus Medical Systems Corp., Tokyo, Japan). Anchors were alternately implanted along opposing full-thickness defect margins. Each anchor was secured by rotating the handle for tissue penetration, followed by depression for release. Following anchor placement in a zigzag pattern across the defect, suture tension was progressively optimized. Traction prompted complete edge approximation, followed by fixation and suture transection using a cinching device, ultimately achieving defect closure ( Fig. 2 ). On postoperative day 1, complete blood count revealed no evidence of delayed hemorrhage. Surveillance endoscopy at one week showed no delayed perforation or bleeding. The six-week endoscopic follow-up revealed the suture anchors retained in situ (partial extrusion) with complete mucosal healing confirmed by biopsy forceps removal ( Fig. 3 ).

**Fig. 2:**
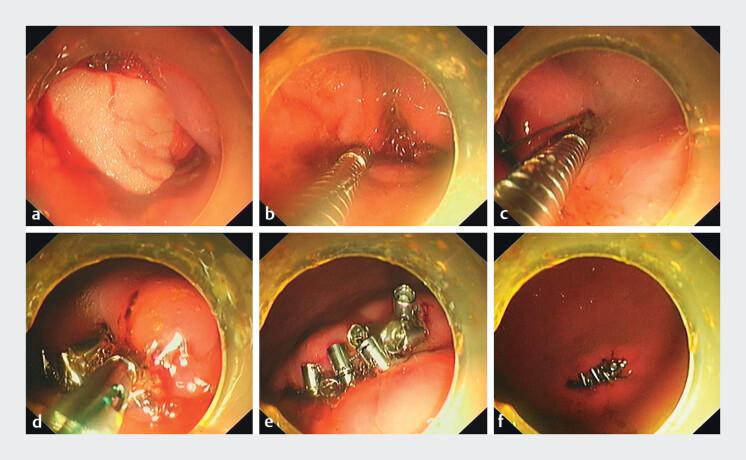
Endoscopic closure of a transmural defect after endoscopic full-thickness resection (EFTR) using novel suture anchors. **a** A simulated post-EFTR transmural defect. **b** Suture-loaded anchor placed 5–10 mm from defect margin. **c** Suture anchors positioned in zigzag pattern. **d** Cinching device advanced with suture. **e** Complete edge approximation preceding suture transection. **f** Closure integrity was verified by gastric insufflation-induced distension.

**Fig. 3:**
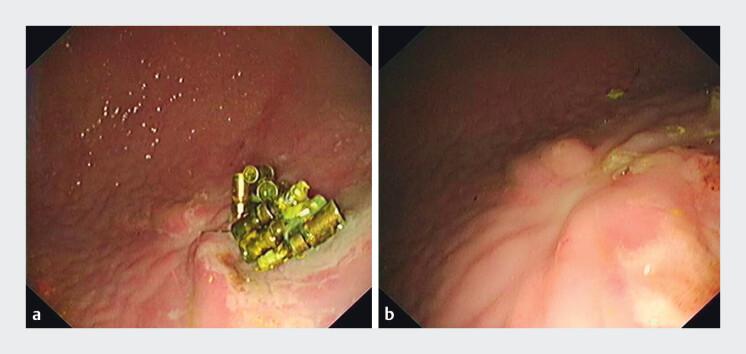
Endoscopic follow-up at postoperative six weeks. **a** Suture anchors retained in situ (partial extrusion). **b** Healing confirmed by forceps removal.

By avoiding superficial grabbing, this suture anchor device facilitates effective closure of full-thickness defects, potentially reducing limitations imposed by defect size. Further studies are required to systematically evaluate its efficacy and safety.

Endoscopy_UCTN_Code_TTT_1AO_2AO

## Notes

Endoscopy E-Videos https://eref.thieme.de/e-videos
*E-Videos* is an open access online section of the journal *Endoscopy* , reporting on interesting cases and new techniques in gastroenterological endoscopy. All papers include a high-quality video and are published with a Creative Commons CC-BY license. Endoscopy E-Videos qualify for HINARI discounts and waivers and eligibility is automatically checked during the submission process. We grant 100% waivers to articles whose corresponding authors are based in Group A countries and 50% waivers to those who are based in Group B countries as classified by Research4Life (see: https://www.research4life.org/access/eligibility/ ). This section has its own submission website at https://mc.manuscriptcentral.com/e-videos .
